# P-50. Mpox Vaccine Uptake Among Sexual and Gender Minority Veterans With and Without HIV in a Nationwide US Veteran Cohort

**DOI:** 10.1093/ofid/ofae631.257

**Published:** 2025-01-29

**Authors:** Puja Van Epps, Wyatt E Meriwether, Alex Mcconnell, Alexis (Lexi) Matza, Jillian Shipherd, Michael R Kauth

**Affiliations:** Veterans Health Administration, Case Western Reserve University School of Medicine, Cleveland, Ohio; Veterans Health Administration, Kansas City, Missouri; Veterans Health Administration, Kansas City, Missouri; Veterans Health Administration, Kansas City, Missouri; Veterans Health Administration/ Boston University, Boston, Massachusetts; Veterans Health Administration/University of Massachusetts, Worcester, Massachusetts

## Abstract

**Background:**

Sexual and gender minorities (SGM) and people with HIV have been disproportionately affected by the Mpox pandemic. Vaccination is recommended for those who may be at an increased risk. We examined the uptake of Mpox vaccine nationally in the Veterans Health Administration (VHA) among SGM Veterans and reported on disparities across groups.

**Figure:**
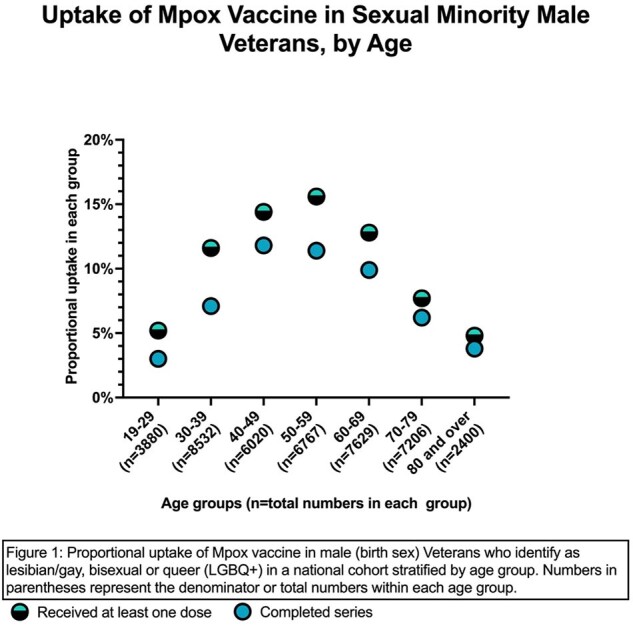

**Methods:**

We queried VHA’s national database to identify Veterans whose sexual orientation is recorded as lesbian, gay, bisexual or queer (LGBQ+) and gender identity as transgender woman (TGW)/man (TGM), non-binary or another gender (except cisgender) [transgender or gender diverse (TGD)] as of April 2024. Birth sex male LGBQ+ and TGD Veterans of both birth sexes were included in the cohort, stratified by age, race/ethnicity and HIV status. Within these cohorts we quantified receipt of JYNNEOS vaccine in the VHA starting 2022.

**Figure:**
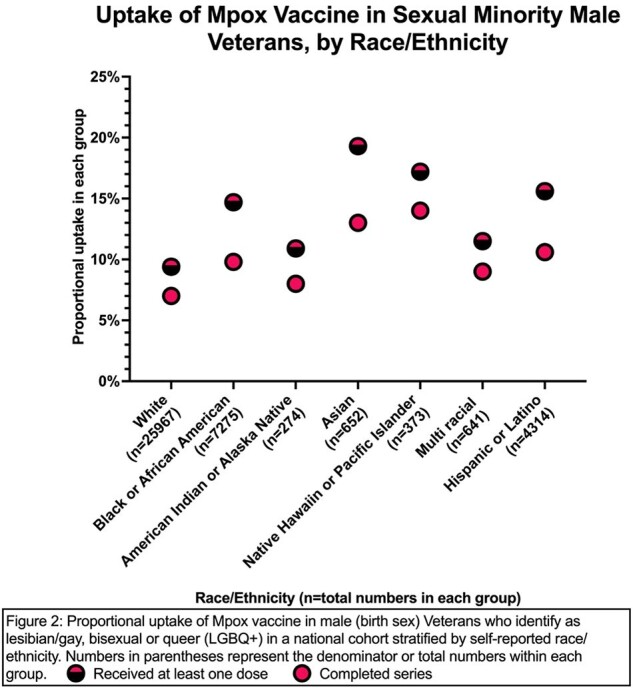

**Results:**

Among the 42,434 males who identify as LGBQ+, 4759 (11.2%) had received at least one dose of the JYNNEOS vaccine; of these, 74% completed the series. There were large variabilities in vaccine uptake among LGBQ+ males by age: the 19-29 cohort had the lowest uptake at 5.2% and lowest series completion rate at 58.1% while the 50-59 age group had the highest uptake at 15.6% (Figure 1). Among racial groups, Asian LGBQ+ males had the highest uptake at 19.3%, and Whites had the lowest uptake at 9.4% (Figure 2). Black and Hispanic LGBQ+ males had better than average uptake at 14.7% and 15.6% respectively. Among 111,338 Veterans who identify as TGD, only 138 (1.2%) had received at least one dose by the end of observation period. Among these, TGW had the highest number of persons vaccinated (n=73) but non-binary Veterans had highest rate at 2.6% (Figure 3). Those with HIV were much more likely to have received at least one dose of the vaccine compared to those without (LGBQ+ males 25.5% vs. 7.6%; TGD 15.1% vs. 1%) (Figure 4).

**Figure:**
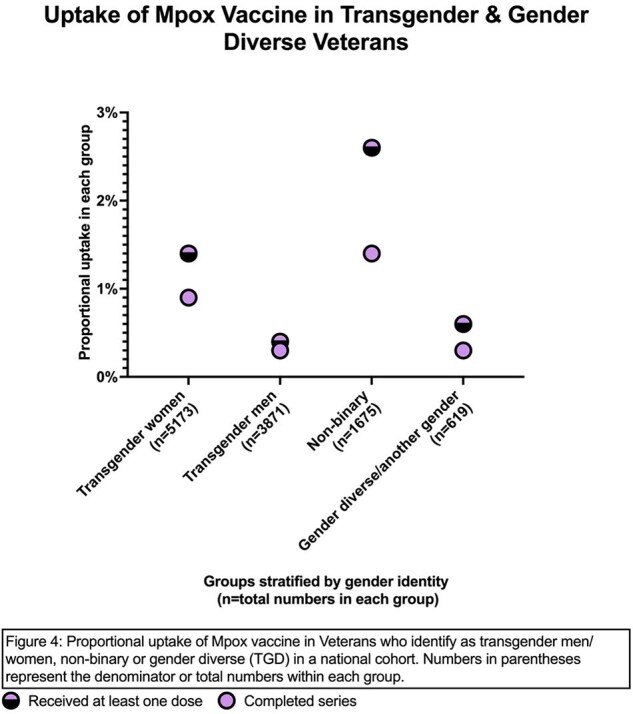

**Conclusion:**

TGD Veterans were much less likely to have received Mpox vaccination in the VHA compared to LGBQ+ males, with HIV status being the strongest predictor of vaccination in both groups. While the overall rates of Mpox vaccination appear lower in the VHA compared to general population, we did not see racial disparities that have been reported in Blacks but observed age related differences.

**Figure:**
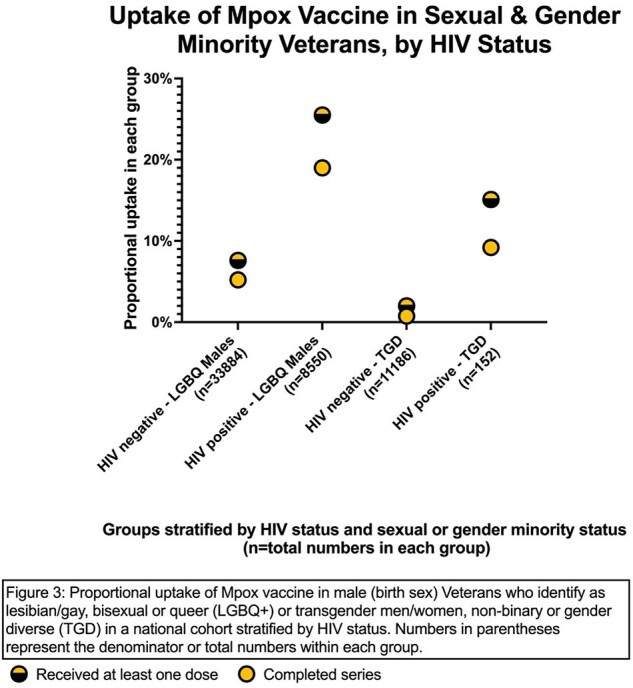

**Disclosures:**

**All Authors**: No reported disclosures

